# News Items

**Published:** 1919-01

**Authors:** 


### NEWS ITEMS.

### Plans for Stopping 1919 Influenza Epidemic.

Plans for combatting another influenza epidemic, which is expected to sweep the country in 1919, were considered by health authorities from all parts of the United States, Canada and South America at the forty-sixth annual convention of the American Public Health Association which was held in Chicago December 9th.

Members of the association say that all the influenza epidemics since 1729 have been recurrent for from two to three years after the initial outbreak. For this reason leading authorities feel convinced that the visitation of 1918 will be repeated in 1919 and probably in 1920. Also it is pointed out that in previous epidemics the second and third outbreaks have been more virulent and attended by a higher mortality rate than were the initial manifestations.

It was early in 1918, according to Dr. W. A. Evans, former health commissioner of Chicago, that the now so-called Spanish influenza made its appearance in Spain. Now Spain is having its second outbreak, according to Dr. Evans more virulent in form and attended by an alarming death rate.

During the three-day meeting, the methods used in combatting the 1918 epidemic in the United States will be thoroughly considered and plans outlined for meeting any future emergency.

---

### Cooties Kill Million.

Of the insects responsible for the deaths or disablement of hundreds of thousands in the war zone, the louse is declared authoritatively to have been one of the most deadly and to accounted for at least 1,000,000 persons.

That, however, is only a rough estimate, and the probability is that the toll was infinitely higher, for in Serbia alone, typhus, a louse-born disease, infected nearly 1,000,000 persons and killed 500 a day in the little city of Jassy, while 200 of the 1,200 medical officers in the country, died from the disease. This disease spread over Russia, Germany and the Balkans generally.

These figures are vouched for in a publication prepared by Lieutenant Lloyd, who was chief entomologist in Northern Rhodesia.

---

Mrs. A. H. Parsons, for the past fourteen years superintendent of the Temple Sanitarium, has resigned that position and will hereafter act in an advisory capacity with that institution. For over twenty years Mrs. Parsons has been identified with hospital work in Temple. Miss Daisy H. Leake has been appointed to succeed her.

---

The new State Hospital for the Negro Insane, constructed about the old penitentiary at Rusk, Texas, is nearing completion and will be ready for patients by April 1, according to Dr. C. L. Gregory of Greenville, superintendent of the institution.

---

### Dr. Gorgas Honored.

In recognition of his distinguished services in behalf of military sanitation, Major-General William C. Gorgas, until recently Surgeon General, United States Army, has been made a grand officer of the Order of the Crown of Italy. The ceremony of presentation took place on November 5, in the office of the Surgeon General, the order being presented by Major General Emilio Guglielmotti, military attache of the Royal Italian Embassy.—Science.

### Flu Takes Big Toll.

Between 300,00 and 350,000 deaths from influenza have occurred among the civilian population of the United States since September 15th, according to estimates given out December 5th by the Public Health Service. These calculations were based on reports from cities and states keeping accurate records and public health officials believe they are conservative.

The epidemic still persists, but deaths are much less numerous, according to reports reaching here. A recrudescence of the disease now is occurring in many communities throughout the country, but this is believed to be sporadic and not to indicate a general renewal of severe epidemic conditions. Insurance companies have been hard hit by the epidemic, government reports indicate, although there are no figures available to show the total losses sustained by the companies.

The government incurred liabilities of more than $170,000,000 in connection with life insurance carried by soldiers in army camps, not including those in Europe. About 20,000 deaths occurred in the camps in the United States, war department records show.

---

Through the generosity of the Tarrant County Medical Society and the trustees for the T. C. U. Medical College, the Carnegie Public Library, is to have one of the largest collections of medical books in the country. The gift of about 1,500 volumes on all subjects relating to medical science was made for the two organizations by Dr. I. R. Chase and Dr. Wilmer Allison. The gift was made possible through the breaking up of the medical college.

---

### Gorgas to Direct Yellow Fever Work.

Dr. William C. Gorgas, who in October retired as surgeon general of the United States army, will resume as director of yellow fever work for the Rockefeller foundation, the work which he temporarily relinquished when this country entered the war. He will sail shortly for South and Central America to direct operations there.

